# Thyrotropin levels in first-episode bipolar disorder and their association with clinical phenotypes: systematic review and meta-analysis

**DOI:** 10.3389/fendo.2025.1580854

**Published:** 2025-08-14

**Authors:** Elisa Gatta, Virginia Maltese, Francesco Dondi, Pietro Bellini, Massimiliano Ugoccioni, Irene Silvestrini, Anna Ceraso, Antonio Vita, Mario Rotondi, Francesco Bertagna, Carlo Cappelli

**Affiliations:** ^1^ Department of Clinical and Experimental Sciences, SSD Endocrinologia, University of Brescia, ASST Spedali Civili, Brescia, Italy; ^2^ Centro per la Diagnosi e Cura delle Neoplasie Endocrine e delle Malattie della Tiroide, University of Brescia, Brescia, Italy; ^3^ Nuclear Medicine, University of Brescia, ASST Spedali Civili, Brescia, Italy; ^4^ Department of Internal Medicine and Therapeutics, University of Pavia, Pavia, Italy; ^5^ Department of Clinical and Experimental Sciences, University of Brescia, ASST Spedali Civili, Brescia, Italy; ^6^ Department of Mental Health and Addiction Services, University of Brescia, ASST Spedali Civili, Brescia, Italy; ^7^ Laboratory for Endocrine Disruptors, Unit of Endocrinology and Metabolism, Istituti Clinici Scientifici Maugeri IRCCS, Pavia, Italy

**Keywords:** thyroid, thyroid hormones, bipolar disorder, TSH, BD manic, BD depression

## Abstract

**Introduction:**

Thyroid hormones play a crucial role in brain function, yet the relationship between TSH and bipolar disorder remains unclear. This review aims to synthesize the current literature to clarify the interplay between serum TSH levels and both the phenotype and severity of bipolar disorder.

**Methods:**

A comprehensive literature search was conducted across PubMed/MEDLINE, Scopus, and Web of Science databases through May 2025. Studies were included based on the PICO framework: What are the TSH levels in first-episode drug-naïve BD patients compared to healthy controls, and do TSH levels differ between manic and depressive phenotypes? The review follows PRISMA guidelines. Study quality and risk of bias were assessed using QUADAS-2.

**Results:**

Seventeen studies out of 3,007 were included. Meta-analysis revealed that 1,946 drug-naïve BD patients had lower TSH levels compared to 400 healthy controls (SMD = -0.395 mIU/L, 95% CI: -0.695 to -0.095). Among 830 BD patients, those with a depressive phenotype (n=494) had higher TSH levels than manic patients (SMD = +0.575 mIU/L, 95% CI: -1.074 to -0.075).

**Discussion:**

Our data suggest that TSH levels can modulate the onset and severity of psychiatric diseases. Interventional studies targeting TSH modulation, particularly in euthyroid patients, are warranted.

## 1 Introduction

Thyroid stimulating hormone (TSH) is a glycoprotein secreted by the anterior pituitary gland, playing a pivotal role in the regulation of thyroid function. It stimulates the synthesis and release of thyroid hormones—primarily thyroxine (T4) and triiodothyronine (T3) (1). TSH secretion is tightly regulated through a negative feedback loop involving circulating T4 and T3 levels, along with hypothalamic release of thyrotropin-releasing hormone (TRH) (2). Because of the sensitivity of this feedback system, variations in TSH are often more pronounced than those in free T4 (fT4), making TSH a sensitive biomarker for detecting fluctuations in thyroid hormone levels (3, 4).

Thyroid hormones (TH) are essential for normal brain function and have been implicated in mood regulation and psychiatric disorders (5). It is well established that TH modulate affective disorders by enhancing serotonergic neurotransmission—specifically by reducing the sensitivity of 5-HT1A autoreceptors in the raphe nuclei and increasing 5-HT2 receptor sensitivity (6). Importantly, thyroid hormone receptors are densely expressed in limbic structures, which are central to mood regulation (7). Additionally, thyronines can bind to several classes of neurotransmitter receptors, including GABAergic, catecholaminergic, glutamatergic, and cholinergic systems (8).

Bipolar disorder (BD) is a complex psychiatric illness characterized by recurrent episodes of mania, hypomania, and depression, with profound effects on mood, energy, and daily functioning (9, 10). BD significantly impairs quality of life and places a considerable burden on healthcare systems globally (10). The estimated lifetime prevalence of BD in adults is 1–3% (11), with a mean onset age of 18–20 years (12), and an approximately equal male-to-female ratio (13). BD is commonly classified into bipolar I disorder—characterized by manic episodes—and bipolar II disorder—characterized by major depressive and hypomanic episodes (14).

Recent evidence suggests that serum TSH levels may influence both the clinical phenotype and the severity of BD (15–24). However, despite the increasing number of studies, the relationship between TSH and BD remains unclear, with conflicting results reported across the literature.

Highlighting a relationship between TSH levels and the clinical presentation or phenotypes of bipolar disorder could have important clinical implications. If confirmed, it could be hypothesized that modulating TSH levels might influence the BD phenotype or presentation, potentially optimizing the response to conventional therapy.

The objective of this systematic review and meta-analysis is to synthesize the available evidence to clarify the relationship between serum TSH levels and the clinical characteristics of BD, including phenotype and severity.

## 2 Materials and methods

### 2.1 Search strategy and inclusion criteria

A wide literature search of the PubMed/MEDLINE, Scopus and Web of Science databases was made.

The review questions were define based on the “Population, Intervention, Comparator, Outcome” framework (PICO): What are the TSH levels in first episode drug-naïve (FEDN) patients diagnosed with bipolar disorder (BD) compared to healthy subjects (comparator), and do TSH levels differ between BD phenotypes (Manic and Depression) (outcome)?

The search algorithm used was (“thyroid”) AND (“bipolar disorder” OR “Bipolar Manic” OR “Bipolar Depression”).

The literature search was updated through May 31, 2025. Only articles in English were considered, and preclinical studies, conference proceedings, reviews, or editorials were excluded. To ensure comprehensive coverage, the reference lists of selected studies were manually screened for additional eligible articles.

### 2.2 Eligibility criteria

The eligibility criteria were chosen taking into account the review question. Clinical studies reporting TSH levels in patients diagnosed with BD were deemed eligible for inclusion in this systematic review. Exclusion criteria for the systematic review (qualitative analysis) were reviews, letters, comments, editorials on the topic of interest, case reports, or small case series (fewer than 5 enrolled patients) on the analyzed topic (as these articles are characterized by poor-quality evidence and are typically affected by publication bias), as well as original articles dealing with different fields of interest.

Both prospective and retrospective observational studies, as well as interventional trials, were considered eligible. This inclusive strategy aimed to optimize data availability while acknowledging the balance between comprehensiveness and methodological rigor.

To avoid confounding due to non-thyroidal illness syndrome (NTIS), only studies reporting the results of thyrotropin-releasing hormone (TRH) test and/or the levels of TSH and thyroid hormone, including free T4 (FT4) and/or T4 and/or free T3 (FT3) and/or T3 in serum or plasma samples were included in the quantitative analysis. In addition, studies involving patients with known thyroid disease were excluded from the meta-analysis.

Studies that addressed the PICO-based review questions were included regardless of the reported TSH values. Euthyroidism was defined based on each study’s specified reference range for serum TSH.

### 2.3 Study selection

E.G. and V.M. independently read the titles and abstracts of the records generated by the search algorithm. They then determined which studies were eligible based on predefined criteria. Any disagreements were resolved through discussion and consensus.

### 2.4 Quality assessment

The quality assessment of these studies, including the risk of bias and applicability concerns, was carried out using Quality Assessment of Diagnostic Accuracy Studies version 2 (QUADAS-2) evaluation (25).

### 2.5 Data extraction

The reviewers collected data from all of the included studies, taking advantage of full-text, tables, and concerning general study information (authors, publication year, country, study design, funding sources), patients’ characteristics (sample size, age, clinical setting, diagnosis, therapies), and TSH levels. The main findings of the articles included in this review are reported in the Results section.

### 2.6 Statistical analysis

The data from the included studies were utilized, considering each study’s relative importance, employing a random-effect statistical model, due to the high heterogeneity in the analyzed studies. Furthermore, the study included the provision of 95% confidence interval values, which were subsequently visually represented through forest plots. The I-square (I^2^) index, also known as the inconsistency index, was employed to assess the level of statistical heterogeneity within the papers included in the analysis. Statistical heterogeneity was considered significant if the I-square index exceeded 50%. The software OpenMeta [Analyst]^®^ (version 3.13), supported by the Agency for Healthcare Research and Quality (AHRQ) in Rockville, MD, USA, was utilized to calculate the pooled values of mean differences.

## 3 Results

### 3.1 Literature search

**Figure 1:**
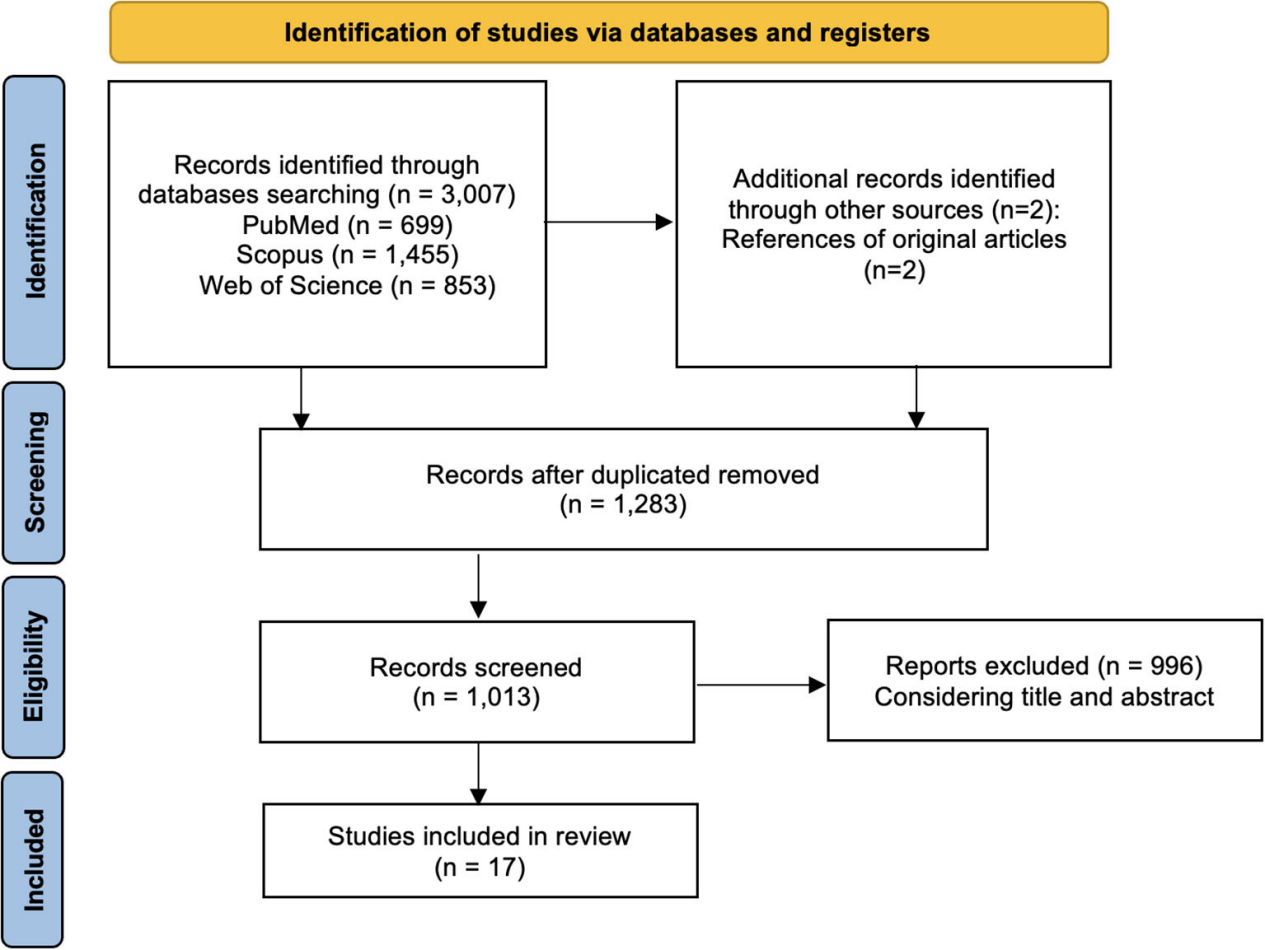
Flowchart of the research of eligible studies on the impact of thyroid function on bipolar disorder.

A total of 3,007 articles were identified through the computer literature search. By reviewing the titles and abstracts, 2,992 articles were excluded because the reported data were not within the field of interest of this review. Consequently, 15 articles were selected and retrieved in full-text versions, and two additional studies were found by screening the references of these articles (**Figure 1**). Therefore, the total number of studies evaluated in the review was 17.

**Figure 2:**
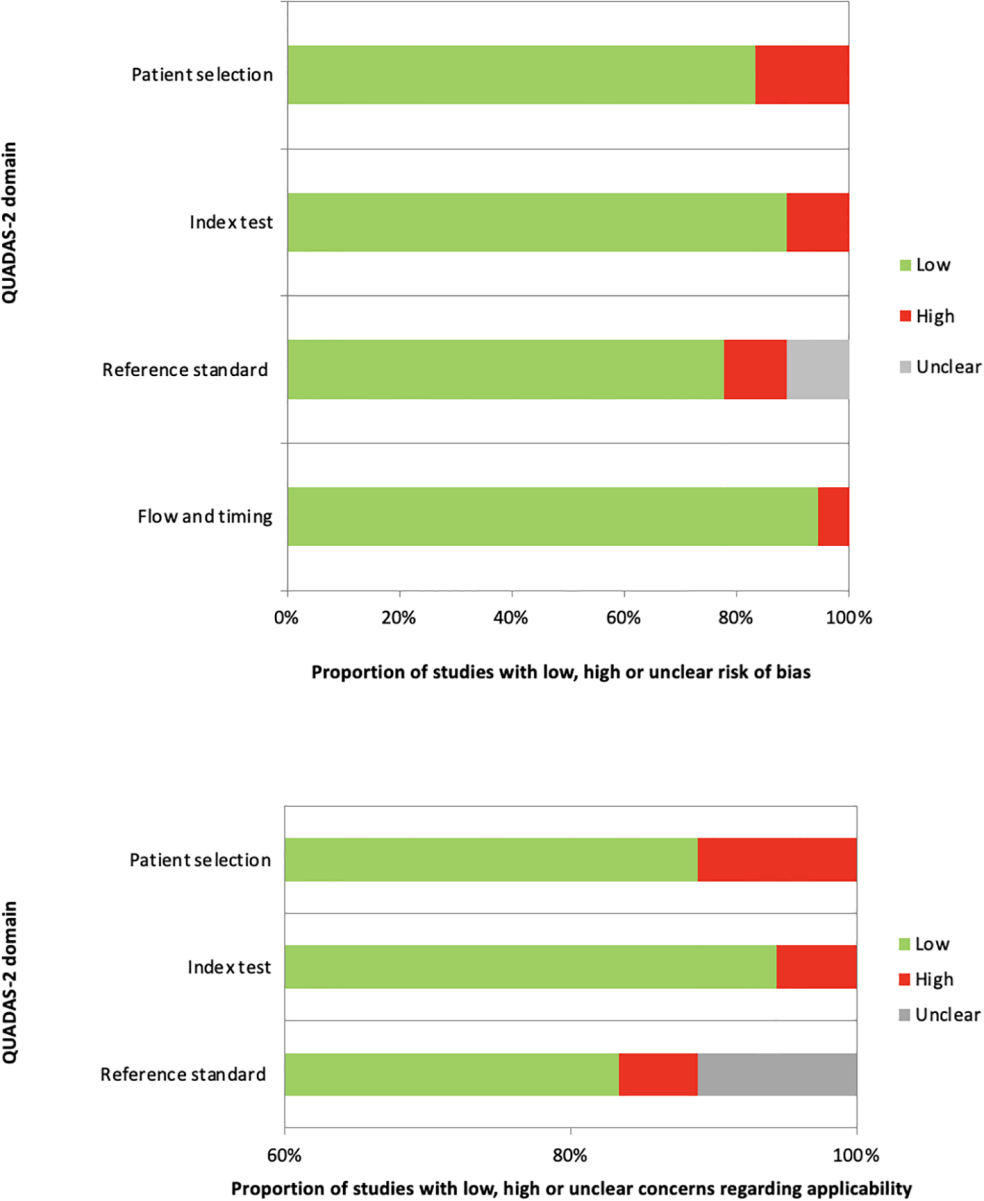
QUADAS-2 quality assessment for risk of bias and applicability concerns for the studies considered in the review. The QUADAS-2 assessment revealed that the most common sources of bias were related to patient selection, including unclear inclusion criteria and retrospective designs, and to flow and timing, particularly with respect to the timing of TSH measurement relative to psychiatric diagnosis.

In general, the quality assessment using QUADAS-2 evaluation underlined the presence of unclear risk of bias and applicability concerns in some of the studies for what concerns patients’ selection, index test, reference standard and flow and timing. Nevertheless, only a small number of studies were characterized by the presence of high risks of bias or applicability (**Figure 2**).

The main characteristics of the studies and their results are briefly presented in **Tables 1** and **2**.

**Table 1:** Characteristics of the human studies considered for the review.

First author	Ref. N.	Year	Country	Study design	N. Pts.	Age	Sex M:F
Extein	(22)	1980	United States	Interventional	10	NA	NA
Kjellman	(15)	1984	Sweden	Prospective cross-sectional	8	54 ± 2.4	4/4
Wahby	(19)	1988	United States	Interventional	14	38.9 ± 3.5	14/0
Wysokiński	(23)	2014	Poland	Retrospective cross-sectional	264	NA	82/182
Degner	(26)	2015	Germany	Prospective cross-sectional	13	42.8	6/7
Li	(27)	2018	China	Prospective cross-sectional	86	23.6 ± 7.4	46/40
Zhong	(20)	2019	China	Prospective cross-sectional	90	26.74 ± 8.73	48/42
Duval	(21)	2021	France	Interventional	13	34.3 ± 10.8	13/0
Lai	(17)	2021	China	Prospective cross-sectional	106	25.45 ± 8.73	49/57
Lieber	(28)	2021	Sweden	Retrospective cohort	291	46.2 (IQR 19.5-87.2)	82/209
Makarow-Gronert	(24)	2021	Poland	Retrospective cross-sectional	32	NA	12/20
Zhao	(29)	2021	China	Prospective cross-sectional	291	27.38 ± 8.01	140/151
Zhu	(30)	2022	China	Retrospective cross-sectional	1333	29.71 ± 9.78	665/668
Chen	(16)	2023	China	Prospective observational	59	23.81 ± 5.87	23/36
Głodek	(31)	2023	Poland	Prospective cross-sectional	54	42.4 ± 13.6	26/28
Song	(18)	2023	China	Prospective cross-sectional	120	30.6 ± 11.2	0/120
Cui	(32)	2024	China	Prospective cross-sectional	1619	29.37 ± 9.78	1049/570

Summary of study design, patient demographics, and clinical setting for the 17 studies included in the review.

Ref., references; N., number; Pts., patients; M, male; F, female; NA, not available.

**Table 2:** Results and main findings of the human studies considered for the review.

First author	Patients’ characteristics	TSH levels (mIU/mL)	TSH reference range (mIU/mL)	Main findings
Chen (16)	Adult inpatients and outpatients with BD aged 18 to 55 years	1.48 ± 0.7	0.49-4.91	BD patients showed significantly lower serum TSH levels compared to healthy controls.
Cui (32)	Drug-free aged below 50 adult patients	1.67 ± 1.64	0.27-4.20	No differences in TSH levels were found among BD and healthy controls
Duval (21)	Drug-free adult male hospitalized patients who underwent TRH-TSH stimulation	1.25 ± 0.58	NA	TSH response is reduced in BDs patients.
Degner (26)	Adult outpatients without previously diagnosed thyroidal diseases.	1.6 ± 1.5	0.4-4.4	No differences in TSH levels were found among BD, MDD and SHZ patients; AbTPO levels were higher in MDD and BD compared to SHZ.
Extein (22)	Consecutive patients diagnosed with BD manic and depression	3.4 ± 0.8	NA	BD depression patients showed a higher TRH-TSH response compared to BD mania subjects.
Głodek (31)	Adults hospitalized in the Department of Adult Psychiatry with age between 18 and 65	1.66 ± 1.17	NA	No significant differences of thyroid function were found between BD and schizophrenia patients.
Kjellman (15)	Adult patients with severe and long-standing clinical history of recurrent episodes of BD	2.9 ± 0.2	NA	No differences in TSH levels were found among BD and healthy controls
Lai (17)	Drug-naïve BD patients aged 17 to 60 years	1.62 ± 0.97	0.49-4.91	BD patients showed significantly lower serum TSH levels compared to healthy controls
Li (27)	Adult drug-naïve BD patients	1.52 ± 0.91	NA	Thyroid functions were not significantly fluctuated between depressive and manic episodes in BD patients
Lieber (28)	Patients having a diagnosis of BD analyzed in the LiSIE (Lithium—Study into Effects and Side Effects) study.	NA	0.27-4.20	Median TSH concentration at the start of THRT was higher in patients treated with lithium than in patients treated with other mood stabilizers. THRT was typically initiated in the context of mild or absent alterations of thyroid function tests with a decreasing TSH threshold.
Makarow-Gronert (24)	Caucasian patients aged 12 to 18 years who were hospitalized in the Department of Adolescent Psychiatry	2.23 ± 1.06	NA	There may be a higher prevalence of thyroid dysfunctions in BD and MDD subgroups among adolescents
Song (18)	First episode, drug-naïve adult female inpatient	2.65 ± 2.39	NA	FT3 levels were significantly lower BD depression patients than healthy controls and higher in BD manic that BD depression.
Wahby (19)	Drug-free washout adult male patients who underwent TRH-TSH stimulation	2.8 ± 0.3	NA	Schizodepressed patients appeared significantly different from MDD but closer to SHZ and healthy controls on the TRH test
Wysokiński (23)	Hospitalized patients in acute phase evaluated at first entry.	1.86 ± 4.58	0.4-5.0	Compared with MDD, patients with BD have the highest level of TSH; BD depression have the highest level of TSH in comparison with BD manic patients.
Zhu (30)	Hospitalized mood disorder patients	2.71 ± 3.27	0.27-4.20	FT4, and FT3 secretion differed between BD and MDD, whereas TSH secretion differed only in the male subgroup
Zhao (29)	Patients diagnosed with BD and who had never received medication	2.29 ± 1.47	0.27-4.20	Compared with BD depression patients, BD manic patients have higher FT3 levels, higher rate of hyperthyroidism and higher rate of total abnormality thyroid hormone secretion.
Zhong (20)	Adult BD inpatients and outpatients diagnosed aged 18 to 55 years.	1.43 ± 0.96	0.38-4.31	BD patients showed significantly lower serum TSH levels compared to healthy controls

Overview of TSH values and key results reported in each study, including comparisons with healthy controls and between bipolar phenotypes.

TSH, thyrotropin stimulating hormone; THRT, thyroid hormones replacement therapy; fT4, free thyroxine; fT3, free triiodothyronine; BD, bipolar disorder; MDD, major depressive disorder; SHZ, schizophrenia; TRH, thyrotropin-releasing hormone.

### 3.2 Qualitative analysis

The available data on 4,403 (51% male) patients affected by BD were retrieved from 17 studies (15–24, 26–32): four were retrospective in nature, ten prospective and three interventional (**Table 1**).

In 1984, Kjelmann et al. showed for the first time in a very small set of euthyroid BD patients a lower serum TSH levels at the onset of disease compared to healthy controls (TSH 2.9 ± 0.2 mIU/L vs 3.6 ± 0.2 mIU/L, p=.026) (15). Subsequent studies confirmed this evidence over forty years of clinical investigations (16–20). On the other hand, recently, Cui et al. did not find any differences between euthyroid BD and healthy subjects (TSH 1.67 ± 1.64 mIU/L vs 1.94 ± 1.28 mIU/L, p=.958) (32). In addition, the only available prospective study, conducted on a small set of euthyroid subjects, confirmed these last data (1.7 ± 1.4 mIU/L vs 1.9 ± 1.5 mIU/L, p=.057) (31).

Going on, Duval et al. showed a reduced TSH response to the TRH test compared to matched healthy control subjects (ΔTSH 1.4 ± 1.3 mIU/L vs 3.8 ± 1.4 mIU/L, p<0.001) (21). Extein et al. confirmed and extended these data in different BD phenotypes (manic and depression) (22).

Wysokiński et al. evaluated TSH serum levels in 1,685 psychiatric inpatients at first entry in a retrospective cross-sectional study also aiming to evaluate any difference between BD phenotypes; BD manic subjects showed lower serum TSH levels than BD depression ones (1.3 ± 1.1 mIU/L vs 2.0 ± 5.2 mIU/L, p=.002) (23). In addition, Makarow-Gronert et al. reinforced these data in a small set of psychiatric euthyroid adolescents (24). By contrast, Li et al. and Zhao et al. did not show any difference in TSH levels between BD manic and depression patients (27, 29).

### 3.3 Quantitative analysis

**Figure 3:**
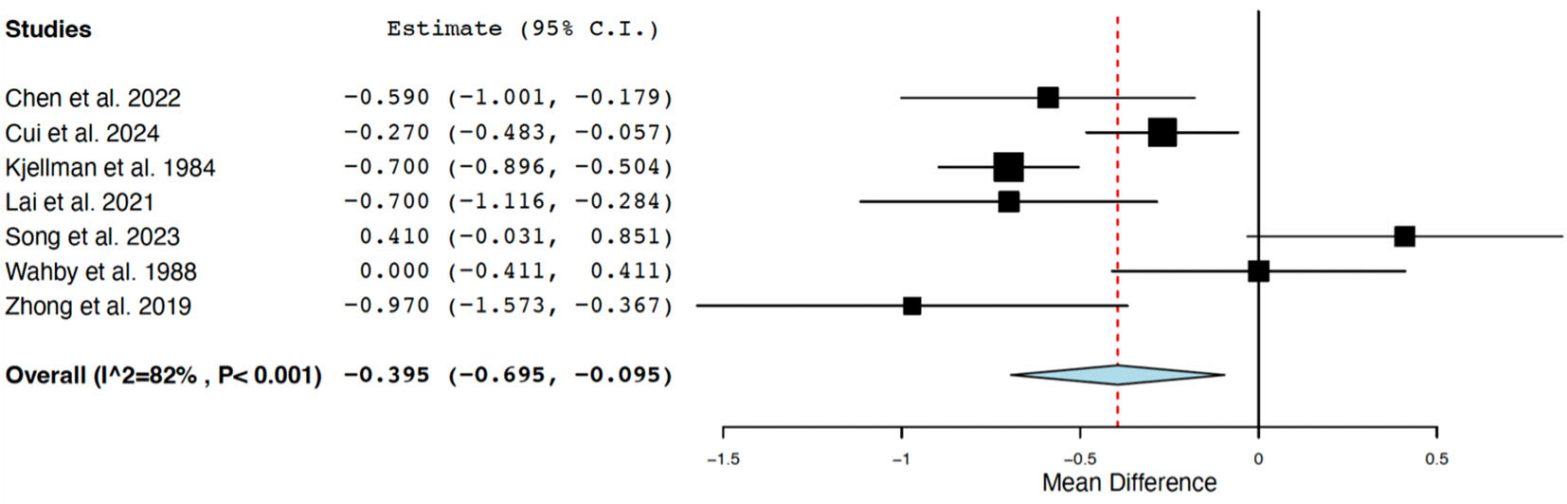
Forest plot of the meta-analysis on serum concentrations of thyroid stimulating hormone (TSH) in patients with first-episode bipolar disorder compared with healthy controls. The figure displays the standardized mean differences (SMDs) in TSH levels between first-episode drug-naïve bipolar disorder patients and healthy controls across seven included studies. Negative values indicate lower TSH levels in bipolar disorder patients.

Seven studies (15–20, 32) comparing TSH levels among BD patients and healthy controls were pooled using the random effect, including 1,946 patients and 400 healthy subjects. One study was from Europe, one from North America and the remaining from Asia; six studies were prospective and one interventional. The meta-analysis showed that FEDN BD patients displayed lower serum TSH levels compared to healthy subjects (SMD=-0.395 mIU/L, C.I. 95%:-0.695 to -0.095) with high heterogeneity across studies (I^2^ = 82%, p<.001) (**Figure 3**).

**Figure 4:**
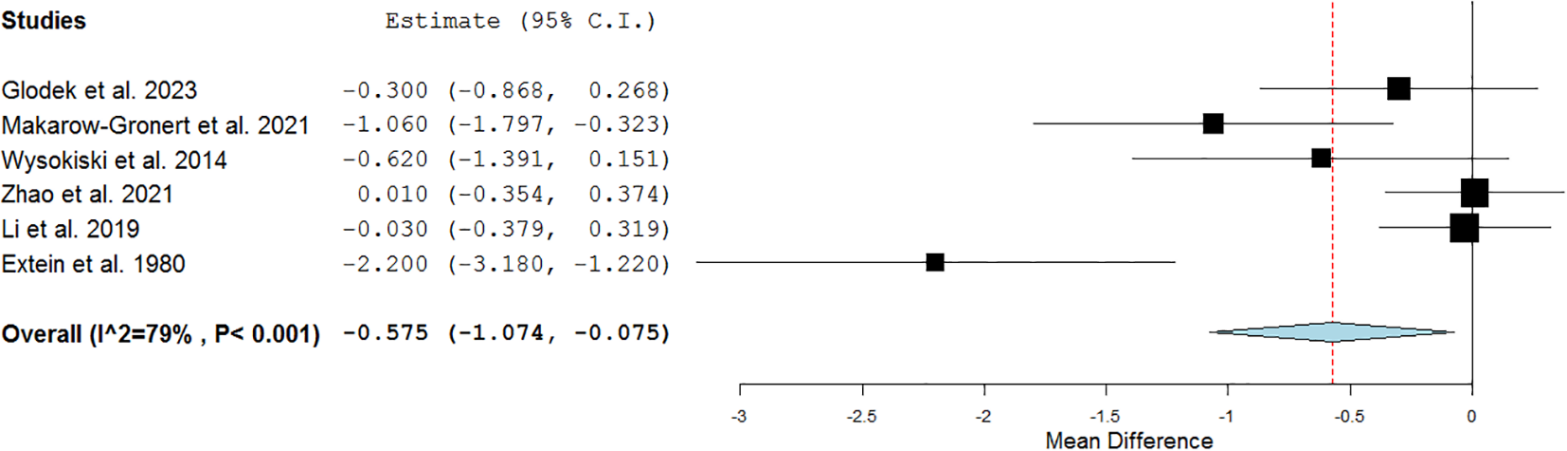
Forest plot of the meta-analysis on serum concentrations of thyroid stimulating hormone (TSH) in patients with first-episode manic bipolar disorder compared with patients with first-episode depressive bipolar disorder. The figure displays the standardized mean differences (SMDs) in TSH levels between manic versus depressive bipolar presentations across six studies. Negative values indicate lower TSH levels in manic patients.

To evaluate any TSH differences among BD manic and BD depressive patients, six studies (22–24, 27, 29, 31) encompassing 830 (336/494 BD manic/depressive) patients were pooled. Two studies were from Asia, three from Europe, and one from North America; two studies were retrospective, three prospective, and one interventional one. The meta-analysis showed that first-episode drug-naïve BD manic patients had lower serum TSH levels compared to BD depressive patients (SMD=-0.575 mIU/L, C.I. 95%: -1.074 to -0.075) with moderate-high heterogeneity across studies (I^2^ = 79%, p<.001) (**Figure 4**).

Both meta-analyses showed substantial heterogeneity (I² > 75%), indicating a high degree of variability among the included studies. Despite the use of a random-effects model to account for this, the pooled effect sizes should be interpreted with caution given the wide dispersion of results.

## 4 Discussion

This study demonstrates that patients with bipolar disorder at the onset of illness exhibit significantly lower serum TSH levels compared to healthy controls. More importantly, variations in thyrotropin concentrations appear to correlate with different BD phenotypes. However, the clinical implications of this finding remain to be fully elucidated.

Mood disorders are multifactorial conditions influenced by complex interactions among various neurotransmitter systems, including norepinephrine, serotonin (5-HT), dopamine, gamma-aminobutyric acid (GABA), and glutamate (33–40). TH are known to play a central role in regulating behavior and mental states, including mood modulation, by modulating the synthesis, release, and metabolism of the aforementioned neurotransmitters (41, 42). Additionally, TH regulates the expression, availability, and activity of 5-HT, DA, and NE transporters, thereby affecting their synaptic concentrations and signaling (42). Disruption of TH balance may result in neurotransmitter dysregulation and mood instability. Furthermore, changes in TH levels can affect neurotransmitter receptor density and affinity, amplifying mood symptoms (43). Animal studies, primarily conducted in mice, have confirmed an association between different emotional behaviors and variations in TSH levels, demonstrating that TSH modulation induces behavioral changes. These studies suggest that TH may contribute to modulating 5-HT uptake and/or GABAergic activity in the hippocampal region and amygdala (44–47).

BD was the 23^rd^ leading cause of years lived with disability in 2016 (48), and patients with BD are more likely to present with coexisting thyroid dysfunction than the general population (49). Duval et al. first reported a reduced TSH response to the TRH stimulation in BD patients compared to healthy controls (21), suggesting potential central dysregulation of thyroid function. However, the only available prospective study, conducted in a small group of euthyroid subjects, found no significant difference in TSH levels between BD patients and healthy controls (31).

Our meta-analysis, which included 2,346 participants across three continents (Europe, North America, and Asia), confirms that patients with newly diagnosed BD have lower serum TSH levels than healthy individuals (**Figure 3**). Moreover, pooled data from 830 patients revealed that those with depressive phenotypes exhibit higher TSH levels than those with manic phenotypes—highlighting a potential endocrine distinction between mood states (**Figure 4**).

Although we used a binary classification of BD into manic and depressive phenotypes, we acknowledge that the disorder encompasses a broader clinical spectrum, including bipolar I and II subtypes, mixed features, and psychotic symptoms. Most included studies did not provide sufficiently detailed diagnostic stratification to allow further subclass analysis. Nevertheless, the consistent TSH differences observed across studies support the notion that thyroid function may influence mood states, even within this simplified framework. Future studies should explore TSH variation in more precisely defined BD subtypes.

Anyway, it is important to underline that the small degree of difference in serum TSH levels found in both meta-analyses could have biological implications, even if it appears to be consistent across many studies. On the other hand, it is well known that there is a log-linear relationship between T3/T4 and TSH, meaning even minor variations in T3/T4 can lead to significant changes in TSH levels (50). Moreover, TSH is highly sensitive to changes in free T3 levels; even subtle fluctuations can trigger significant changes in TSH. This supports the role of TSH, in the absence of hypothalamic/pituitary disease, as the most sensitive marker of thyroid function (51). Since brain function is dependent on T3 availability, TSH variation may indirectly reflect shifts in T3 concentrations.

Understanding the connection between BD and TH requires elucidating how this interplay, along with the aforementioned effects on neurotransmitters, influences BD symptoms. Animal models have shown that TH infusion increases 5-HT levels in the rodent brain cortex (6). Many studies have been conducted to evaluate the impact of TH modulation on BD management. Bauer et al., in a randomized, placebo-controlled, double-blinded trial, demonstrated the effectiveness of high-dose levothyroxine (L-T4) in mitigating depressive symptoms in euthyroid individuals with bipolar depression (52). These findings were further supported by an open-label study in women with treatment-resistant BD depression, which also reported changes in positron emission tomography (PET) features, reflecting altered metabolism in the prefrontal, subcortical, and limbic regions (53). These results have been corroborated by placebo-controlled studies (54–56), and treatment with supraphysiological doses of L-T4 is now recommended in several BD management guidelines (57–60). If validated, our findings could contribute to a more personalized approach to BD management. For instance, pharmacological modulation of TSH might be considered for BD I versus BD II patients, depending on phenotype-specific thyroid profiles. Furthermore, many guidelines recommend routine TSH screening in mood disorder evaluations (61–64). However, how this finding impacts real medical practice remains to be demonstrated. In other words, can treatment with L-T4 or thioamide influence the behavior of different BD phenotypes? What are the optimal TSH levels for BD patients? Is it time to consider personalizing TSH targets based on BD phenotypes? Currently, no global data are currently available on euthyroid BD depressive patients treated with minimal doses of L-T4 or thioamides aiming at modulating TSH levels while maintaining them within the normal range; further studies are necessary to address this gap.

Several limitations of the present study must be acknowledged. The most notable limitation is the substantial statistical heterogeneity observed in both meta-analysis (I² > 75%) which reflects considerable clinical and/or methodological variability across studies. This undermines the strength of the pooled estimates and limits the generalizability of the findings. Although we used a random-effects model, the underlying sources of heterogeneity remain only partially explained. Additionally, there is a significant heterogeneity in the time intervals between BD diagnosis and TSH measurement. While the studies included in this review and meta-analysis were of good quality, the small sample sizes introduce a greater margin of error and an increased risk of bias. Finally, another key limitation is the heterogeneity in study design, which included retrospective, prospective, and interventional studies carrying an additional risk of bias. This factor, combined with the high heterogeneity, limits the overall certainty of the findings.

Finally, it is important to underline that a limitation of this review is the lack of a pre-registered protocol. Although the methodology was defined *a priori* by all authors, the absence of a publicly accessible protocol may limit the transparency and reproducibility of the review process.

## 5 Conclusion

We have reviewed the literature from the past forty years regarding the possible interplay between thyroid function and BD patients. Our meta-analysis evidenced that different thyrotropin levels are associated to different BD phenotype. If and how this issue could have an impact on general practice is still to be assessed. Interventional studies aimed to modulate TSH values are needed.

## Data Availability

The original contributions presented in the study are included in the article/Supplementary Material. Further inquiries can be directed to the corresponding author.

## References

[1] Persani L . Clinical review: Central hypothyroidism: pathogenic, diagnostic, and therapeutic challenges. J Clin Endocrinol Metab. (2012) 97:3068–78. doi: 10.1210/jc.2012-1616, PMID: 22851492

[2] Biondi B Cooper DS . The clinical significance of subclinical thyroid dysfunction. Endocr Rev. (2008) 29:76–131. doi: 10.1210/er.2006-0043, PMID: 17991805

[3] Fitzgerald SP Bean NG . Thyroid stimulating hormone (TSH) autoregulation reduces variation in the TSH response to thyroid hormones. Temperature (Austin). (2018) 5:380–9. doi: 10.1080/23328940.2018.1513110, PMID: 30574530 PMC6298488

[4] Chaker L Bianco AC Jonklaas J Peeters RP . Hypothyroidism. Lancet. (2017) 390:1550–62. doi: 10.1016/S0140-6736(17)30703-1, PMID: 28336049 PMC6619426

[5] Bauer M Goetz T Glenn T Whybrow PC . The thyroid-brain interaction in thyroid disorders and mood disorders. J Neuroendocrinol. (2008) 20:1101–14. doi: 10.1111/j.1365-2826.2008.01774.x, PMID: 18673409

[6] Bauer M Heinz A Whybrow PC . Thyroid hormones, serotonin and mood: of synergy and significance in the adult brain. Mol Psychiatry. (2002) 7:140–56. doi: 10.1038/sj.mp.4000963, PMID: 11840307

[7] Bauer M London ED Silverman DH Rasgon N Kirchheiner J Whybrow PC . Thyroid, brain and mood modulation in affective disorder: insights from molecular research and functional brain imaging. Pharmacopsychiatry. (2003) 36 Suppl 3:S215–21. doi: 10.1055/s-2003-45133, PMID: 14677082

[8] Martin JV Sarkar PK . Nongenomic roles of thyroid hormones and their derivatives in adult brain: are these compounds putative neurotransmitters? Front Endocrinol (Lausanne). (2023) 14:1210540. doi: 10.3389/fendo.2023.1210540, PMID: 37701902 PMC10494427

[9] ICD-11 for Mortality and Morbidity Statistics. World Health Organization (WHO. Available online at: http://id.who.int/icd/entity/334423054 (Accessed January 31, 2025).

[10] Grande I Berk M Birmaher B Vieta E . Bipolar disorder. Lancet. (2016) 387:1561–72. doi: 10.1016/S0140-6736(15)00241-X, PMID: 26388529

[11] Pedersen CB Mors O Bertelsen A Waltoft BL Agerbo E McGrath JJ . A comprehensive nationwide study of the incidence rate and lifetime risk for treated mental disorders. JAMA Psychiatry. (2014) 71:573–81. doi: 10.1001/jamapsychiatry.2014.16, PMID: 24806211

[12] Merikangas KR Jin R He JP Kessler RC Lee S Sampson NA . Prevalence and correlates of bipolar spectrum disorder in the world mental health survey initiative. Arch Gen Psychiatry. (2011) 68:241–51. doi: 10.1001/archgenpsychiatry.2011.12, PMID: 21383262 PMC3486639

[13] Seedat S Scott KM Angermeyer MC Berglund P Bromet EJ Brugha TS . Cross-national associations between gender and mental disorders in the World Health Organization World Mental Health Surveys. Arch Gen Psychiatry. (2009) 66:785–95. doi: 10.1001/archgenpsychiatry.2009.36, PMID: 19581570 PMC2810067

[14] Diagnostic and statistical manual of mental disorders (5th ed., text rev.). American Psychiatric Association (2022).

[15] Kjellman BF Beck-Friis J Ljunggren JG Wetterberg L . Twenty-four-hour serum levels of TSH in affective disorders. Acta Psychiatr Scand. (1984) 69:491–502. doi: 10.1111/j.1600-0447.1984.tb02524.x, PMID: 6741599

[16] Chen P Chen G Zhong S Chen F Ye T Gong J . Thyroid hormones disturbances, cognitive deficits and abnormal dynamic functional connectivity variability of the amygdala in unmedicated bipolar disorder. J Psychiatr Res. (2022) 150:282–91. doi: 10.1016/j.jpsychires.2022.03.023, PMID: 35429738

[17] Lai S Zhong S Zhang Y Wang Y Zhao H Chen G . Association of altered thyroid hormones and neurometabolism to cognitive dysfunction in unmedicated bipolar II depression. Prog Neuropsychopharmacol Biol Psychiatry. (2021) 105:110027. doi: 10.1016/j.pnpbp.2020.110027, PMID: 32791168

[18] Song X Feng Y Yi L Zhong B Li Y . Changes in thyroid function levels in female patients with first-episode bipolar disorder. Front Psychiatry. (2023) 14:1185943. doi: 10.3389/fpsyt.2023.1185943, PMID: 38025417 PMC10679747

[19] Wahby VS Ibrahim GA Giller EL Mason JW Saddik FW Adams JR . Thyrotropin response to thyrotropin-releasing hormone in RDC schizodepressed men. J Affect Disord. (1988) 15:81–5. doi: 10.1016/0165-0327(88)90012-2, PMID: 2970496

[20] Zhong S Chen G Zhao L Jia Y Chen F Qi Z . Correlation between intrinsic brain activity and thyroid-stimulating hormone level in unmedicated bipolar II depression. Neuroendocrinology. (2019) 108:232–43. doi: 10.1159/000497182, PMID: 30673659

[21] Duval F Mokrani MC Erb A Danila V Gonzalez Lopera F Jeanjean L . Dopaminergic, noradrenergic, adrenal, and thyroid abnormalities in psychotic and affective disorders. Front Psychiatry. (2020) 11:533872. doi: 10.3389/fpsyt.2020.533872, PMID: 33101075 PMC7546351

[22] Extein I Pottash AL Gold MS Cadet J Sweeney DR Davies RK . The thyroid-stimulating hormone response to thyrotropin-releasing hormone in mania and bipolar depression. Psychiatry Res. (1980) 2:199–204. doi: 10.1016/0165-1781(80)90077-3, PMID: 6774358

[23] Wysokiński A Kłoszewska I . Level of thyroid-stimulating hormone (TSH) in patients with acute schizophrenia, unipolar depression or bipolar disorder. Neurochem Res. (2014) 39:1245–53. doi: 10.1007/s11064-014-1305-3, PMID: 24723220 PMC4103998

[24] Makarow-Gronert A Margulska A Strzelecki D Krajewska K Gmitrowicz A Gawlik-Kotelnicka O . Comparison of thyroid-stimulating hormone levels in adolescents with schizophrenia, bipolar disorder, unipolar depression, conduct disorders, and hyperkinetic disorders. Med (Baltimore). (2021) 100:e28160. doi: 10.1097/MD.0000000000028160, PMID: 34889284 PMC8663859

[25] Whiting PF Rutjes AW Westwood ME Mallett S Deeks JJ Reitsma JB . QUADAS-2: a revised tool for the quality assessment of diagnostic accuracy studies. Ann Intern Med. (2011) 155:529–36. doi: 10.7326/0003-4819-155-8-201110180-00009, PMID: 22007046

[26] Degner D Haust M Meller J Rüther E Reulbach U . Association between autoimmune thyroiditis and depressive disorder in psychiatric outpatients. Eur Arch Psychiatry Clin Neurosci. (2015) 265:67–72. doi: 10.1007/s00406-014-0529-1, PMID: 25193677

[27] Li C Lai J Huang T Han Y Du Y Xu Y . Thyroid functions in patients with bipolar disorder and the impact of quetiapine monotherapy: a retrospective, naturalistic study. Neuropsychiatr Dis Treat. (2019) 15:2285–90. doi: 10.2147/NDT.S196661, PMID: 31496710 PMC6691940

[28] Lieber I Ott M Öhlund L Lundqvist R Eliasson M Sandlund M . Patterns of thyroid hormone prescription in patients with bipolar or schizoaffective disorder: findings from the LiSIE retrospective cohort study. J Clin Med. (2021) 10:5062. doi: 10.3390/jcm10215062, PMID: 34768582 PMC8584539

[29] Zhao S Zhang X Zhou Y Xu H Li Y Chen Y . Comparison of thyroid function in different emotional states of drug-naïve patients with bipolar disorder. BMC Endocr Disord. (2021) 21:210. doi: 10.1186/s12902-021-00869-5, PMID: 34674686 PMC8532266

[30] Zhu Y Ji H Niu Z Liu H Wu X Yang L . Biochemical and endocrine parameters for the discrimination and calibration of bipolar disorder or major depressive disorder. Front Psychiatry. (2022) 13:875141. doi: 10.3389/fpsyt.2022.875141, PMID: 35795028 PMC9251015

[31] Głodek M Skibinska M Suwalska A . Diet and physical activity and metabolic disorders in patients with schizophrenia and bipolar affective disorder in the Polish population. PeerJ. (2023) 11:e15617. doi: 10.7717/peerj.15617, PMID: 37456885 PMC10348314

[32] Cui T Qi Z Wang M Zhang X Wen W Gao S . Thyroid allostasis in drug-free affective disorder patients. Psychoneuroendocrinology. (2024) 162:106962. doi: 10.1016/j.psyneuen.2024.106962, PMID: 38277991

[33] Ressler KJ Nemeroff CB . Role of norepinephrine in the pathophysiology and treatment of mood disorders. Biol Psychiatry. (1999) 46:1219–33. doi: 10.1016/S0006-3223(99)00127-4, PMID: 10560027

[34] Dremencov E el Mansari M Blier P . Brain norepinephrine system as a target for antidepressant and mood stabilizing medications. Curr Drug Targets. (2009) 10:1061–8. doi: 10.2174/138945009789735165, PMID: 19702556

[35] Kurita M . Noradrenaline plays a critical role in the switch to a manic episode and treatment of a depressive episode. Neuropsychiatr Dis Treat. (2016) 12:2373–80. doi: 10.2147/NDT.S109835, PMID: 27703355 PMC5036557

[36] Kato T . Current understanding of bipolar disorder: Toward integration of biological basis and treatment strategies. Psychiatry Clin Neurosci. (2019) 73:526–40. doi: 10.1111/pcn.12852, PMID: 31021488

[37] Mansour HA Talkowski ME Wood J Pless L Bamne M Chowdari KV . Serotonin gene polymorphisms and bipolar I disorder: focus on the serotonin transporter. Ann Med. (2005) 37:590–602. doi: 10.1080/07853890500357428, PMID: 16338761

[38] Stone JM Davis JM Leucht S Pilowsky LS . Cortical dopamine D2/D3 receptors are a common site of action for antipsychotic drugs–an original patient data meta-analysis of the SPECT and PET *in vivo* receptor imaging literature. Schizophr Bull. (2009) 35:789–97. doi: 10.1093/schbul/sbn009, PMID: 18303092 PMC2696370

[39] Bielau H Steiner J Mawrin C Trübner K Brisch R Meyer-Lotz G . Dysregulation of GABAergic neurotransmission in mood disorders: a postmortem study. Ann N Y Acad Sci. (2007) 1096:157–69. doi: 10.1196/annals.1397.081, PMID: 17405927

[40] Shen J Tomar JS . Elevated brain glutamate levels in bipolar disorder and pyruvate carboxylase-mediated anaplerosis. Front Psychiatry. (2021) 12:640977. doi: 10.3389/fpsyt.2021.640977, PMID: 33708149 PMC7940766

[41] Krishna VN Thunga R Unnikrishnan B Kanchan T Bukelo MJ Mehta RK . Association between bipolar affective disorder and thyroid dysfunction. Asian J Psychiatr. (2013) 6:42–5. doi: 10.1016/j.ajp.2012.08.003, PMID: 23380316

[42] Alcaide Martin A Mayerl S . Local thyroid hormone action in brain development. Int J Mol Sci. (2023) 24:12352. doi: 10.3390/ijms241512352, PMID: 37569727 PMC10418487

[43] Seshadri A Sundaresh V Prokop LJ Singh B . Thyroid hormone augmentation for bipolar disorder: A systematic review. Brain Sci. (2022) 12:1540. doi: 10.3390/brainsci12111540, PMID: 36421864 PMC9688441

[44] Schneider MJ Fiering SN Pallud SE Parlow AF St Germain DL Galton VA . Targeted disruption of the type 2 selenodeiodinase gene (DIO2) results in a phenotype of pituitary resistance to T4. Mol Endocrinol. (2001) 15:2137–48. doi: 10.1210/mend.15.12.0740, PMID: 11731615

[45] Bárez-López S Montero-Pedrazuela A Bosch-García D Venero C Guadaño-Ferraz A . Increased anxiety and fear memory in adult mice lacking type 2 deiodinase. Psychoneuroendocrinology. (2017) 84:51–60. doi: 10.1016/j.psyneuen.2017.06.013, PMID: 28654773

[46] Venero C Guadaño-Ferraz A Herrero AI Nordström K Manzano J de Escobar GM . Anxiety, memory impairment, and locomotor dysfunction caused by a mutant thyroid hormone receptor alpha1 can be ameliorated by T3 treatment. Genes Dev. (2005) 19:2152–63. doi: 10.1101/gad.346105, PMID: 16131613 PMC1221886

[47] Maglione AV do Nascimento BPP Ribeiro MO de Souza TJL da Silva REC Sato MA . Triiodothyronine Treatment reverses Depression-Like Behavior in a triple-transgenic animal model of Alzheimer’s Disease. Metab Brain Dis. (2022) 37:2735–50. doi: 10.1007/s11011-022-01055-9, PMID: 35951206

[48] Global, regional, and national incidence, prevalence, and years lived with disability for 328 diseases and injuries for 195 countries, 1990-2016: a systematic analysis for the Global Burden of Disease Study 2016. Lancet. (2017) 390:1211–59. doi: 10.1016/S0140-6736(17)32154-2, PMID: 28919117 PMC5605509

[49] Smith DJ Martin D McLean G Langan J Guthrie B Mercer SW . Multimorbidity in bipolar disorder and undertreatment of cardiovascular disease: a cross sectional study. BMC Med. (2013) 11:263. doi: 10.1186/1741-7015-11-263, PMID: 24359325 PMC3880052

[50] Pirahanchi Y Toro F Jialal I . Physiology, Thyroid Stimulating Hormone. StatPearls. Treasure Island (FL: StatPearls Publishing Copyright © 2025, StatPearls Publishing LLC (2025). 29763025

[51] Sheehan MT . Biochemical testing of the thyroid: TSH is the best and, oftentimes, only test needed - A review for primary care. Clin Med Res. (2016) 14:83–92. doi: 10.3121/cmr.2016.1309, PMID: 27231117 PMC5321289

[52] Bauer M Berman S Stamm T Plotkin M Adli M Pilhatsch M . Levothyroxine effects on depressive symptoms and limbic glucose metabolism in bipolar disorder: a randomized, placebo-controlled positron emission tomography study. Mol Psychiatry. (2016) 21:229–36. doi: 10.1038/mp.2014.186, PMID: 25600111 PMC4790155

[53] Bauer M London ED Rasgon N Berman SM Frye MA Altshuler LL . Supraphysiological doses of levothyroxine alter regional cerebral metabolism and improve mood in bipolar depression. Mol Psychiatry. (2005) 10:456–69. doi: 10.1038/sj.mp.4001647, PMID: 15724143

[54] Stamm TJ Lewitzka U Sauer C Pilhatsch M Smolka MN Koeberle U . Supraphysiologic doses of levothyroxine as adjunctive therapy in bipolar depression: a randomized, double-blind, placebo-controlled study. J Clin Psychiatry. (2014) 75:162–8. doi: 10.4088/JCP.12m08305, PMID: 24345793

[55] Walshaw PD Gyulai L Bauer M Bauer MS Calimlim B Sugar CA . Adjunctive thyroid hormone treatment in rapid cycling bipolar disorder: A double-blind placebo-controlled trial of levothyroxine (L-T(4)) and triiodothyronine (T(3)). Bipolar Disord. (2018) 20:594–603. doi: 10.1111/bdi.12657, PMID: 29869405 PMC6323302

[56] Pilhatsch M JS T Stahl P Lewitzka U Berghöfer A Sauer C . Treatment of bipolar depression with supraphysiologic doses of levothyroxine: a randomized, placebo-controlled study of comorbid anxiety symptoms. Int J Bipolar Disord. (2019) 7:21. doi: 10.1186/s40345-019-0155-y, PMID: 31583561 PMC6776578

[57] Grunze H Vieta E Goodwin GM Bowden C Licht RW Möller HJ . The World Federation of Societies of Biological Psychiatry (WFSBP) Guidelines for the Biological Treatment of Bipolar Disorders: Update 2010 on the treatment of acute bipolar depression. World J Biol Psychiatry. (2010) 11:81–109. doi: 10.3109/15622970903555881, PMID: 20148751

[58] Practice guideline for the treatment of patients with bipolar disorder (revision). Am J Psychiatry. (2002) 159:1–50. 11958165

[59] Sachs GS Printz DJ Kahn DA Carpenter D Docherty JP . The expert consensus guideline series: medication treatment of bipolar disorder 2000. Postgrad Med. (2000). 1–104. 10895797

[60] Yatham LN Kennedy SH Parikh SV Schaffer A Bond DJ Frey BN . Canadian Network for Mood and Anxiety Treatments (CANMAT) and International Society for Bipolar Disorders (ISBD) 2018 guidelines for the management of patients with bipolar disorder. Bipolar Disord. (2018) 20:97–170. doi: 10.1111/bdi.12609, PMID: 29536616 PMC5947163

[61] National Institute for Health and Care Excellence: Guidelines . Bipolar disorder: assessment and management. London: National Institute for Health and Care Excellence (NICE) Copyright © NICE 2024 (2023). 31556980

[62] Kato T Ogasawara K Motomura K Kato M Tanaka T Takaesu Y . Practice guidelines for bipolar disorder by the JSMD (Japanese society of mood disorders). Psychiatry Clin Neurosci. (2024) 78:633–45. doi: 10.1111/pcn.13724, PMID: 39194164 PMC11804931

[63] Garber JR Cobin RH Gharib H Hennessey JV Klein I Mechanick JI . Clinical practice guidelines for hypothyroidism in adults: cosponsored by the American Association of Clinical Endocrinologists and the American Thyroid Association. Thyroid. (2012) 22:1200–35. doi: 10.1089/thy.2012.0205, PMID: 22954017

[64] Vanderpump MP Ahlquist JA Franklyn JA Clayton RN . Consensus statement for good practice and audit measures in the management of hypothyroidism and hyperthyroidism. The Research Unit of the Royal College of Physicians of London, the Endocrinology and Diabetes Committee of the Royal College of Physicians of London, and the Society for Endocrinology. Bmj. (1996) 313:539–44. doi: 10.1136/bmj.313.7056.539, PMID: 8789985 PMC2351923

